# Advances in Pharmacological Approaches to Tinnitus and Hyperacusis: Insights into Mechanisms, Biomarkers, and Clinical Heterogeneity from an International Scientific Meeting

**DOI:** 10.3390/brainsci16050450

**Published:** 2026-04-24

**Authors:** Hashir Aazh, Vinay Parameshwarappa, Arnaud Noreña, Kelly N. Jahn, Stefan Fink, Stephan Wolpert, Rodrigo Donoso-San Martín, Lukas Rüttiger, Marlies Knipper, Wei Sun, Richard Salvi

**Affiliations:** 1 Hashir International Institute, London W1W 5PF, UK; 2 Centre de Recherche en Psychologie et Neurosciences, Aix-Marseille Université, 13003 Marseille, France; vinayparameshwarappa.ir@gmail.com (V.P.); arnaud.norena@univ-amu.fr (A.N.); 3 Department of Speech, Language, and Hearing, The University of Texas at Dallas, Richardson, TX 75080, USA; kelly.jahn@utdallas.edu; 4 Callier Center for Communication Disorders, Dallas, TX 75235, USA; 5 Department of Otolaryngology—Head and Neck Surgery, Hearing Cognition and Tinnitus, Molecular Physiology of Hearing, University of Tübingen, 72076 Tübingen, Germany; stefan.fink@medizin.uni-tuebingen.de (S.F.); stephanwolpert@gmx.net (S.W.); rodrigo-andres.donoso-san-martin@uni-tuebingen.de (R.D.-S.M.); lukas.ruettiger@uni-tuebingen.de (L.R.); marlies.knipper@uni-tuebingen.de (M.K.); 6 Laboratorio de Neurobiología de la Audición, Department of Neuroscience, Universidad de Chile, Santiago 8320000, Chile; 7 Department of Communicative Disorders and Sciences, State University of New York at Buffalo, Buffalo, NY 14260, USA; weisun@buffalo.edu; 8 Department of Communication Disorders and Sciences, University of Central Florida, Orlando, FL 32816, USA; salvi@buffalo.edu

**Keywords:** tinnitus, hyperacusis, pharmacology, pain hyperacusis

## Abstract

**Highlights:**

**What are the main findings?**

- Tinnitus and hyperacusis are heterogeneous conditions involving multiple mechanisms and patient presentations.
- Mechanistic research is advancing understanding but has not yet translated into disease-modifying pharmacological treatments.

**What are the implications of the main findings?**

- Clinical assessment and management may benefit from a stratified approach that considers individual risk factors and symptom profiles.
- Mechanistic insights may improve patient education and personalised care while current treatments remain focused on coping and quality of life.

**Abstract:**

Pharmacological approaches to tinnitus have remained peripheral within research, despite persistent patient demand and advances in auditory neuroscience. There is increasing consideration of whether these developments now justify renewed focus on pharmacological research in tinnitus and hyperacusis. This paper presents a narrative synthesis of perspectives arising from an international scientific meeting, based on invited contributions from speakers who expanded on themes related to their presentations, integrating insights from systems neuroscience, cellular and molecular mechanisms, animal and human models of auditory hypersensitivity, and patient-reported experience. Rather than summarising individual presentations, the analysis focuses on converging mechanistic explanations, biological heterogeneity, emerging therapeutic targets, and advances in objective measurement. Particular attention is given to how evolving mechanistic frameworks can support patient education and clinical communication, even in the absence of disease-modifying treatments. Taken together, these discussions reflect renewed examination of pharmacological approaches to tinnitus and hyperacusis, with attention to both emerging mechanistic rationales and the substantial challenges that remain. Integrating mechanistic insights, phenotype-informed care, and responsible communication may help clarify research priorities and support informed clinical and patient-facing discussions.

## 1. Introduction

Research into tinnitus has long been characterised by a productive tension between mechanism and meaning. On the one hand, tinnitus is investigated as a neurophysiological phenomenon, emerging from altered activity across the auditory periphery and central nervous system. On the other, it is experienced as a deeply personal and life-altering condition for some people, one that challenges assumptions about suffering, adaptation, and the goals of medical intervention. The Second Conference on Pharmacology and Gene Therapy for Tinnitus was conceived within this tension, not merely as a technical meeting, but as a forum for reflecting on why and how pharmacological research in tinnitus should proceed.

While contemporary tinnitus conferences routinely include sessions on drug trials or molecular targets, pharmacology has typically remained one strand among many. Yet surveys consistently show that for many individuals with tinnitus, the prospect of a medical treatment that could suppress, modify, or prevent tinnitus remains a primary priority [1]. This expectation persists despite decades of pharmacological investigation across both animal models and clinical contexts, where numerous compounds have been explored without demonstrating robust and reproducible effects in clinical settings, as highlighted in several comprehensive reviews [2]. In the absence of a reliable cure, pharmacological approaches in clinical practice have largely focused on managing associated symptoms, with medications such as tricyclic antidepressants and benzodiazepines used off-label to address distress, anxiety, or sleep disturbance, although their effects on tinnitus perception itself remain variable and, in some cases, may exacerbate symptoms [3]. This creates a philosophical and ethical question that extends beyond feasibility: should the field accept coping-based interventions as sufficient, or does it retain an obligation to pursue biological treatments even in the face of complexity and uncertainty?

This question framed much of the conceptual structure of the conference. At its core lies an inquiry into causation. Does tinnitus arise primarily from aberrant signalling in the cochlea and auditory nerve, from maladaptive plasticity in central auditory pathways, or from distributed network-level processes involving thalamic, limbic, and cortical systems? If tinnitus reflects measurable changes in neuronal activity, can these changes serve as reliable physiological markers suitable for pharmacological targeting and treatment validation? Addressing such questions requires not only molecular and cellular insight, but also advances in imaging, signal analysis, and translational methodology.

Equally important is the recognition that tinnitus does not exist in isolation. Related forms of sound intolerance, including loudness hyperacusis, pain hyperacusis, and misophonia, arise across diverse contexts such as noise exposure, pharmacological manipulation, stress, and neurodevelopmental conditions, and raise parallel questions concerning neural excitability, inhibitory control, and sensory gain. These conditions challenge any single-mechanism account and instead suggest that similar clinical symptoms may emerge from distinct biological processes. The conference therefore adopted a deliberately integrative perspective, moving from synapses and ion channels to patient experience, and from molecular targets to lived disability.

Underlying these scientific discussions was a broader normative perspective. Drawing on Aristotelian thought, a distinction can be made between addressing immediate needs and pursuing longer-term aims related to human flourishing [4]. In this context, coping-based therapies remain essential, as they respond directly to the immediate needs of individuals experiencing tinnitus. By contrast, pharmacological research represents a longer-term endeavour, aimed at engaging with underlying biological mechanisms and the possibility of disease modification. From a wider ethical standpoint, such research can be understood as a commitment to reducing future burden through sustained scientific inquiry. The Second Conference on Pharmacology and Gene Therapy for Tinnitus was therefore positioned not only as a scientific exchange, but also as a collective effort to advance understanding and care.

Rather than summarising individual presentations, this paper presents a narrative synthesis of conference contributions, drawing on invited speakers who expanded on topics related to their presentations. It highlights shared mechanistic perspectives, emerging pharmacological targets, methodological advances, and ongoing clinical challenges in tinnitus and hyperacusis. In bringing together these perspectives, the conference reaffirmed that pharmacological research in tinnitus is neither redundant nor naïve, but a necessary complement to existing therapies, grounded in scientific responsibility and ethical purpose.

## 2. Disrupted Inhibitory Control and Central Gain as Candidate Mechanisms

Several contributions explored tinnitus and hyperacusis through the lens of altered inhibitory regulation and changes in central auditory gain. Within this broad perspective, attention was drawn to the contrast between congenital and acquired deafness. It was noted that tinnitus is reported infrequently in congenital deafness, even when hearing loss is profound or unilateral, whereas it is commonly associated with hearing loss acquired later in life [5,6].

This observation was discussed not only in mechanistic terms, but also in relation to a more fundamental question: whether tinnitus depends on the presence of specific neural activity, or on the recognition and reportability of that activity. If similar forms of internally generated auditory activity were present from birth, they may never be identified as tinnitus, remaining phenomenologically unformed or indistinguishable from the baseline of experience. In contrast, when auditory experience has developed and is subsequently altered, internally generated signals may be more readily recognised as aberrant and thus reported as tinnitus. From this perspective, the relative absence of tinnitus reports in congenital deafness does not necessarily indicate the absence of tinnitus-related neural activity, but may instead reflect differences in perceptual reference, experiential contrast, and the conditions required for conscious recognition. This interpretation aligns with broader considerations regarding the epistemic status of tinnitus, namely that it becomes accessible as tinnitus through recognition and, in many cases, through report.

At the same time, mechanistic interpretations were also considered. One proposed account [7] emphasises developmental differences in inhibitory organisation within the auditory system, suggesting that auditory experience contributes to the maturation of temporal processing through increased tonic parvalbumin-positive interneuron (PV-IN) inhibitory strength, known to play a crucial role in cortical microcircuit formation [8,9]. Within this framework, weakening of high-spontaneous-rate auditory fibre input later in life, for example, following cochlear synaptopathy or stress exposure, may reduce sustained inhibitory control across frequency channels, as suggested for cortical plasticity changes in somatosensory or auditory systems [10]. Poor sustained inhibition following weakening of high-spontaneous-firing-rate fibres could potentially contribute to increased neural excitability and altered thalamocortical dynamics [7]. However, the extent to which such mechanisms fully account for the observed differences with regard to tinnitus experience between congenital and acquired deafness remains unresolved.

Within this interpretative framework, altered inhibition has been suggested as a potential bridge between models, emphasising increased spontaneous activity, reduced sensory input, and predictive or inferential processes [11]. Rather than resolving these perspectives, this account offers one way of relating them through shared assumptions about gain regulation and network stability [12]. Alternative accounts place greater emphasis on cortical network dynamics, attention, learning, or non-auditory modulatory systems, underscoring the absence of a single unifying model.

Evidence from animal studies further illustrates the complexity of central gain mechanisms. Chronic moderate-level noise exposure has been shown to induce persistent hypersensitivity and increased neural excitation in subcortical and cortical auditory structures without measurable threshold shifts [13]. While such findings demonstrate that central auditory plasticity can occur independently of “visible” peripheral damage (assessed through Auditory Brainstem Response measurements), their relevance to subjective tinnitus and hyperacusis in humans remains an area of active investigation rather than settled conclusion [14]. It is also assumed that certain lasting functional or structural changes may occur at the peripheral and central levels due to the temporary threshold shift induced by noise exposure, while cochlear damage may resolve completely after a few days.

## 3. Cellular and Molecular Mechanisms of Inhibitory Failure

At the cellular level, several experimental studies have examined mechanisms through which inhibitory regulation within auditory pathways may be altered following acoustic trauma, potentially shifting adaptive plasticity toward maladaptive outcomes [15,16,17]. One line of investigation has focused on disruption of chloride homeostasis in auditory neurons.

Inhibitory neurotransmission mediated by GABA depends on maintaining low intracellular chloride concentrations, a process regulated in part by the potassium chloride cotransporter KCC2 [18]. Animal studies have shown that intense noise exposure can reduce KCC2 expression in auditory structures such as the dorsal cochlear nucleus and inferior colliculus, leading to intracellular chloride accumulation [19]. Under such conditions, GABAergic signalling may lose its hyperpolarising effect and become depolarising, a phenomenon previously characterised in other neurological contexts, including neuropathic pain [20]. Consistent with this interpretation, functional recordings in noise-exposed animals have shown that pharmacological blockade of GABA receptors can paradoxically reduce sound-evoked firing, suggesting a reversal of inhibitory polarity under these experimental conditions [19]. These findings provide a plausible cellular mechanism for increased auditory gain and sound intolerance in animal models. However, their relevance to subjective hyperacusis and tinnitus in humans remains uncertain.

Building on this evidence, discussions during the meeting focused on the potential role of KCC2 downregulation, which has been observed in cases of severe noise-induced hearing loss. A key question that emerged was the threshold at which such changes occur, specifically the minimum level of acoustic trauma or degree of hearing loss required to alter KCC2 expression. Participants also considered whether combining KCC2 enhancers with GABA agonists could amplify inhibitory function and offer a more targeted approach to reducing neuronal hyperactivity. At the same time, concerns were raised regarding the lack of selectivity of such pharmacological strategies, with the potential to broadly alter central neural activity. Pharmacodynamic habituation was also highlighted as a possible limitation, potentially reducing long-term efficacy.

Related molecular insights have emerged from genetic models associated with auditory hypersensitivity. In Fragile X syndrome, altered glutamatergic signalling involving metabotropic glutamate receptor 5 (mGluR5) has been linked to exaggerated loudness perception despite normal auditory thresholds [21]. In animal models, pharmacological inhibition of mGluR5 with MTEP has been shown to normalise behavioural measures of loudness, illustrating that targeted intervention can modify hyperacusis-like phenotypes when a specific molecular mechanism is implicated [21]. As with other preclinical findings, translation to broader clinical populations remains uncertain.

## 4. Heterogeneity of Hyperacusis: Noise, Stress, Neurodevelopment, and Pain

Hyperacusis was discussed as a heterogeneous condition encompassing multiple phenotypes that may arise from distinct biological pathways rather than a single underlying mechanism. Contributions addressed several partially overlapping explanatory frameworks, including noise-related models emphasising compensatory central gain, stress-related modulation of auditory processing, neurodevelopmental mechanisms (genetic conditions) involving altered cortical adaptation, and pain hyperacusis associated with nociceptive and trigeminal pathways.

Noise-related forms of hyperacusis are often interpreted within frameworks emphasising compensatory central gain following reduced or altered peripheral input [22,23]. By contrast, other forms of sound intolerance have been observed in the absence of measurable cochlear injury, suggesting that peripheral damage is not a necessary condition for symptom development.

Experimental studies have demonstrated that elevation of stress hormones such as corticosterone can induce exaggerated loudness perception and sound avoidance behaviour in animal models, accompanied by increased activity in the auditory cortex without detectable cochlear dysfunction [24]. These findings suggest that stress-related modulation of central auditory processing may contribute to variability and fluctuation in sound intolerance, although their relevance to chronic symptoms in humans remains to be clarified.

Neurodevelopmental models further illustrate mechanistic diversity. In animal models of Fragile X syndrome and FoxG1 mutation, sound hypersensitivity has been associated with impaired cortical adaptation rather than overt hyperexcitability [21,25]. Related alterations in auditory cortical adaptation have also been reported in autistic individuals, indicating potential convergence across genetic and developmental contexts [26].

A particularly severe phenotype is pain hyperacusis, also referred to as noxacusis. Qualitative and survey-based studies have described sound-evoked pain as burning, stabbing, or throbbing, often radiating beyond the ear and frequently co-occurring with reactive tinnitus [27,28]. Several mechanisms have been proposed to account for this presentation, including activation of cochlear type II afferents with nociceptor-like properties [29], middle-ear muscle dysfunction involving trigeminal pathways [30,31], and processes related to central sensitisation. The relative contribution of these mechanisms remains an open question.

## 5. Measurement and Validation: Toward Objective Biomarkers

Progress in pharmacological research for tinnitus and hyperacusis is often constrained by the limited availability of objective, mechanism-sensitive outcome measures. A range of methodological approaches have been explored to address this challenge, particularly in preclinical and translational research contexts.

High-resolution neuroimaging techniques, including magnetencephalography (MEG) and optically pumped magnetometer magnetoencephalography (OPM-MEG), have been proposed as potential tools for tinnitus research for differentiating spontaneous and stimulus-evoked neural activity across oscillatory frequency bands [32]. Such methods may offer improved sensitivity to neural dynamics relevant to auditory perception, although their role as biomarkers for tinnitus or hyperacusis remains under investigation.

Behavioural assays, such as reaction-time-based measures of loudness growth and active sound avoidance paradigms, have provided quantitative indices of auditory hypersensitivity in animal models [33]. In human studies, pupillometry has been examined as a potential objective correlate of auditory sensitivity and listening effort, with the advantage of scalability across laboratory, clinical, and remote assessment settings [25]. As with other candidate measures, further validation is required to establish specificity, reliability, and relevance to patient-reported outcomes.

## 6. Clinical Reality and the Ethical Imperative for Pharmacology

Despite advances in mechanistic understanding, patient-reported data underscore the limitations of current treatments, particularly for pain hyperacusis and severely distressing tinnitus. Individuals frequently report profound disability and limited benefit from existing behavioural and sound-based interventions [27,34].

Survey data indicate that patients often experiment with a wide range of pharmacological agents with highly variable outcomes, highlighting the limitations of trial-and-error approaches in the absence of validated targets and biomarkers. From an ethical standpoint, this pattern underscores the need for more rigorous and targeted pharmacological research as a complement to existing therapeutic approaches.

## 7. Implications for Tinnitus Education and Patient Communication

Beyond their relevance for research, mechanistic models of tinnitus and hyperacusis may also influence how these conditions are explained to patients. Several concepts discussed in the literature have been proposed as potentially useful for patient communication, provided they are presented with appropriate attention to uncertainty and individual variability.

One such concept is the framing of tinnitus and hyperacusis as conditions involving altered neural regulation rather than damage alone. Observations that tinnitus is rarely reported in congenital deafness but commonly follows hearing loss acquired later in life have been interpreted as suggesting that tinnitus is part of an experience-dependent maturation step involving fast auditory neuronal processing that develops only after hearing onset [7]. When communicated carefully, this distinction may help patients contextualise their symptoms without equating tinnitus with progressive neural degeneration.

Related explanatory models emphasise changes in central gain and inhibitory balance within auditory pathways. Describing tinnitus as reflecting increased neural sensitivity in response to reduced or distorted input has been proposed as a way to link patient experience with experimental and computational models of auditory processing [22,23]. Such accounts may also help explain the frequent co-occurrence of sound intolerance among individuals seeking help for tinnitus, including reduced uncomfortable loudness levels and heightened auditory sensitivity during clinical assessment, as commonly reported in specialist clinic populations [35]. Importantly, these models are compatible with symptom variability over time, including fluctuations associated with stress, fatigue, or sound exposure.

Experimental findings linking stress-related hormones to changes in auditory sensitivity further highlight the relevance of psychophysiological factors without implying that tinnitus is primarily psychological in origin. Evidence that chronic stress can influence central auditory gain and cortical excitability [24] has been interpreted as providing a biological context for addressing stress, sleep, and autonomic regulation as part of broader tinnitus care, rather than as stand-alone explanations.

Recognition of heterogeneity is also central to patient education. Evidence suggesting that loudness hyperacusis and pain hyperacusis may arise through different mechanisms such as altered gain regulation, impaired cortical adaptation, or engagement of nociceptive pathways has implications for how advice and expectations are communicated [27]. Acknowledging this diversity may help explain why standard recommendations are not uniformly effective and reduce the risk of patients interpreting limited benefit as personal failure.

Finally, emerging work on objective measures, including pupillometry and neuroimaging, illustrate ongoing efforts to characterise tinnitus and hyperacusis using quantifiable neural markers. While such tools are not yet suitable for routine clinical use, their cautious inclusion in educational discussions may help convey that these conditions are being actively investigated as measurable neurobiological phenomena, without overstating their current applicability.

Overall, these perspectives suggest that patient education can draw on scientific discoveries to provide explanations that emphasise regulation, plasticity, and individual variability, while remaining transparent about current uncertainties and limitations. Such an approach may support informed discussion and realistic expectations without implying definitive mechanisms or treatments.

## 8. Clinical Implications

Mechanistic research in tinnitus and hyperacusis may have implications for clinical practice even in the absence of disease-modifying pharmacological treatments. One recurring theme in the literature is the importance of recognising heterogeneity. Evidence from experimental, clinical, and patient-reported studies indicates that tinnitus and hyperacusis do not represent unitary conditions, suggesting that clinical assessment may benefit from considering factors such as noise exposure history, stress vulnerability, neurodevelopmental background, pain symptoms, and patterns of sound reactivity.

This heterogeneity has implications for management strategies. Interventions that are helpful for some presentations may be ineffective or poorly tolerated in others. For example, exposure-based sound therapies have been reported as beneficial in certain forms of loudness hyperacusis but may exacerbate symptoms in individuals with pain hyperacusis or suspected nociceptive mechanisms [27]. Similarly, where symptoms show strong fluctuation in relation to stress or arousal, it may be reasonable to integrate stress-modulating approaches earlier in care, informed by experimental evidence linking stress hormones to changes in central auditory gain [24].

Experimental studies of inhibitory dysregulation and gain alteration also raise questions about timing. Preclinical data suggest that maladaptive plasticity can develop over days to weeks following acoustic trauma, indicating a potential window during which central changes become consolidated [19]. While these findings do not translate directly into specific pharmacological recommendations, they reinforce the clinical value of early assessment, careful patient education, and avoidance of prolonged sound deprivation, which has been proposed as a factor that may further destabilise inhibitory control.

From a pharmacological perspective, identification of molecular targets such as KCC2 and mGluR5 reflects progress in understanding candidate mechanisms rather than readiness for clinical application. In this context, transparent communication is essential. Clinicians may need to balance discussion of emerging research with clear emphasis on current treatment goals, which remain focused on reducing distress, functional impairment, and disability while disease-modifying therapies continue to be investigated. Patient survey data indicate that access to clear explanations about tinnitus and hyperacusis, including discussion of proposed mechanisms and treatment rationales, is consistently valued and associated with greater treatment satisfaction [36,37,38,39].

Developments in objective outcome measures also have potential implications for future research and service development. Behavioural paradigms, pupillometry, and advanced neuroimaging have been explored as complements to self-report measures, particularly in populations for whom subjective rating scales are challenging, such as children and individuals with neurodevelopmental conditions. Any transition toward multimodal assessment frameworks is likely to be gradual and contingent on further validation.

Overall, existing evidence supports a cautious, stratified approach to clinical care that acknowledges mechanistic diversity while remaining grounded in current therapeutic realities. Established interventions aimed at supporting coping and quality of life remain central. At the same time, emerging mechanistic insights may inform patient education, refine individualised management, and provide context for future developments without implying immediate changes to clinical practice.

## 9. Conclusions

This paper synthesises current perspectives on pharmacological research in tinnitus and hyperacusis, highlighting the diversity of mechanisms, methodological challenges, and translational uncertainties in the field. These conditions are increasingly conceptualised as disorders of dysregulated auditory and neural systems, with variable trial outcomes likely reflecting underlying heterogeneity rather than absence of biological relevance. Mechanistic insights may inform clinical understanding even in the absence of effective treatments, while ethical considerations remain central, with coping-based approaches addressing current distress, and pharmacological research oriented toward longer-term possibilities. Overall, progress will depend on interdisciplinary collaboration, careful interpretation of evidence, and clear communication, with pharmacological research forming one part of a broader, integrative approach to care.

## Data Availability

No new data were created or analysed in this study. Data sharing is not applicable to this article.
